# The Role of the CDK4/6 Inhibitor Ribociclib in Locally Advanced and Oligometastatic Hormone Receptor Positive, Her2 Negative, Advanced Breast Cancer: Case Series and Review of the Literature

**DOI:** 10.3389/fonc.2022.797157

**Published:** 2022-02-09

**Authors:** Andrea Botticelli, Agnese Fabbri, Michela Roberto, Daniele Alesini, Alessio Cirillo, Giuliana D’Auria, Eriseld Krasniqi, Eleonora Marrucci, Margherita Muratore, Francesco Pantano, Laura Pizzuti, Ilaria Portarena, Rosalina Rossi, Simone Scagnoli, Paolo Marchetti

**Affiliations:** ^1^ Medical Oncology Unit B, Policlinico Umberto I, Rome, Italy; ^2^ Department of Clinical and Molecular Medicine, Sapienza University of Rome, Oncology Unit, Sant’Andrea Hospital, Rome, Italy; ^3^ Medical Oncology Unit, Belcolle Hospital, Viterbo, Italy; ^4^ Medical Oncology Unit A, Policlinico Umberto I, Rome, Italy; ^5^ UOSD Centro Oncologico S. Spirito e Nuovo Regina Margherita (SS-NRM), Ospedale Santo Spirito in Sassia, Rome, Italy; ^6^ Department of Radiological, Oncological and Anatomo-Pathological Science, “Sapienza” University of Rome, Rome, Italy; ^7^ Department of Medical Oncology, Sandro Pertini Hospital, Rome, Italy; ^8^ Division of Medical Oncology 2, Istituti di Ricovero e Cura a Carattere Scientifico (IRCCS) Regina Elena National Cancer Institute, Rome, Italy; ^9^ Division of Gynecologic Oncology, Department of Woman and Child Health and Public Health, Fondazione Policlinico Universitario Agostino Gemelli Istituti di Ricovero e Cura a Carattere Scientifico (IRCCS), Roma, Italy; ^10^ Department of Oncology, University Campus Biomedico of Rome, Rome, Italy; ^11^ Medical Oncology Unit, Internal Medicine Department, Tor Vergata Clinical Center University Hospital, Rome, Italy; ^12^ Medical Oncology, San Giovanni Addolorata Hospital, Rome, Italy; ^13^ Department of Medical and Surgical Sciences and Translational Medicine, “Sapienza” University of Rome, Rome, Italy

**Keywords:** CDK4/6 inhibitor, oligometastatic, locally advanced breast cancer (LABC), ribociclib, case report

## Abstract

The recent addition of cyclin-dependent kinase 4 (CDK4) and CDK6 inhibitors to endocrine therapy has remarkably improved the outcome of patients affected with hormone receptor positive (HR+), human epidermal grow factor receptor 2 negative (HER2 -) advanced breast cancer (ABC). Ribociclib showed to be effective across most subgroups, regardless of the number and the site of metastasis. Up to 10% of patients with ABC, reported an oligometastatic condition, recently defined as a slow-volume metastatic disease with limited number and size of metastatic lesions (up to 5 and not necessarily in the same organ), potentially amenable for local treatment, aimed at achieving a complete remission status. Despite the wide use of CDK4/6 inhibitors in HR+, HER2-, ABC treatment, data regarding both locally advanced, inoperable disease and oligometastatic conditions are still poor. We reported a review and case series of HR+, HER2-, ABC patients treated with ribociclib as first-line therapy, for a locally advanced and oligometastatic conditions, reporting an impressive response and good safety profile.

## Introduction

The recent addition of cyclin-dependent kinase 4 (CDK4) and CDK6 inhibitors to endocrine therapy has remarkably improved the outcome of patients affected with hormone receptor positive (HR+), human epidermal grow factor receptor 2 negative (HER2 -) advanced breast cancer (ABC) (1). To date, three third-generation CDK4/6 inhibitors (palbociclib, ribociclib, abemaciclib) have been approved in combination with aromatase inhibitor (AI) or fulvestrant, in both first and subsequent lines of therapy, according to phase III trial results (2–7).

All three of these CDK4/6 inhibitors demonstrated comparable results in terms of response rate, progression-free survival (PFS), and overall survival (OS), especially if administered in combination with fulvestrant (8–10). However, at ESMO 2021, a statistically significant and clinically meaningful OS benefit with ribociclib and letrozole in postmenopausal patients with HR+/HER2– ABC was reported for the first time (11).

Moreover, a pooled analysis of patient-reported quality of life in the MONALEESA-2, -3, and -7 trials of ribociclib plus ET also reported a great impact in terms of delayed deterioration of global health status, pain, and emotional functioning (12), suggesting that ribociclib plus ET is not only effective but also safe.

The survival benefit of ribociclib was consistent across most subgroups, regardless of the number and site of metastases (8, 9). In 1% to 10% of patients with ABC occurs an oligometastatic condition, recently defined as a slow volume metastatic disease with limited number and size of metastatic lesions (up to 5 and not necessarily in the same organ), potentially amenable for local treatment (1). Despite the wide use of CDK4/6 inhibitors in HR+, HER2-, ABC treatment, data regarding both locally advanced, inoperable disease and oligometastatic conditions are still poor. We reported a case series of HR+, HER2-, ABC patients treated with ribociclib as first-line therapy, in two cases for a locally advanced, inoperable disease and the other two cases for oligometastatic conditions, reporting an impressive response and good safety profile.

## Patients and Method

Clinical records of four patients affected with HR+, HER2-, ABC, treated with ribociclib as first-line therapy, in two different settings were reviewed. The first and second cases involve patients treated with fulvestrant 500 mg d1,28 plus ribociclib 600 mg/die d 1,21 q 28 for a locally advanced, inoperable disease, whereas the other two cases concern patients affected with ABC treated with letrozolo 2.5 mg/die plus ribociclib 600 mg/die d1,21 q28 for an oligometastatic disease. Tumor response was determined according to the Response Evaluation Criteria in Solid Tumors (RECIST), version 1.1. The severity of adverse events was graded according to CTCAE version 4.0. All patients signed an informed consent to therapy and research purposes at the time of the first oncological visit.

## Results

### Case Report 1: Patient With a Locally Advanced, Inoperable, Recurrent Disease

The first case involved a 63-year-old patient, in good general condition (performance status, ECOG PS = 0), who had menarche at 11 years, physiological menopause at the age of 52 years, no pregnancy and/or abortion, and no comorbidities.

**Figure 1:**
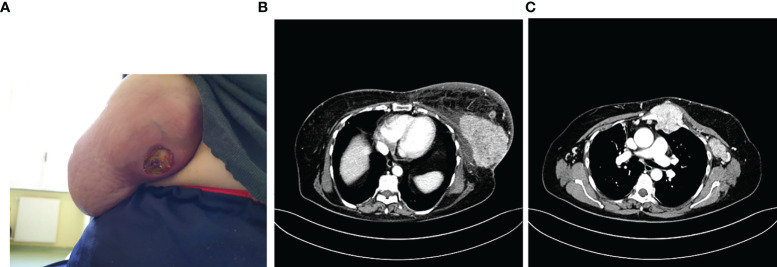
Photo of the ulcerated swelling at the level of the external quadrants of the left breast **(A)**, described also at the CT scan **(B)** with the involvement of locoregional lymph nodes and sternum **(C)**.

In 2010, she underwent left quadrantectomy surgery plus sentinel lymph node biopsy (SNLB) for a moderately differentiated invasive ductal carcinoma (IDC), non-specific type (NST), with ER 98%, PR 80%, Ki-67 10%, HER-2 negative, immunohistochemistry (IHC) profile (stage pT1c pN0). Therefore, she was subjected to adjuvant treatment with radiotherapy (RT) and hormone therapy (HT) with letrozole 2.5 mg for 5 years. Follow-up for relapse of disease was negative until May 2020 when it came to our attention for the first time, reporting from about 15 days of the onset of an ulcerated swelling at the level of the left breast (**Figure 1A**). A bilateral mammary ultrasound revealed a significant increase in the volume of the left breast, with the presence at the superior external quadrant of a hypoechoic, heterogeneous, and intensely vascularized large solid mass, of 8 cm in the maximum diameter. Some homolateral axillary lymph-adenopathies of 4 cm in diameter were also identified. The biopsy reported the presence of IDC, NST, poorly differentiated (G3), with ER 98%, PR 25%, ki67: 45%, HER-2 negative, IHC expression. PIK3CA on the circulating tumor DNA was also performed with no evidence of mutation. The CT scan described the presence of a solid mass of 10 × 6 cm at the level of the external superior left quadrants (QQEE) and nearby, another one of 12 × 14 mm. Also, the presence of a solid lesion along the vessels of the left internal mammary chain (55 × 53 cm), with necrotic-colliquative appearance, which infiltrates the ribs, sternum, intercostal muscles, and large pectoral, was reported. Further lymph nodes, with similar characteristics, are observed in the left axilla (50 × 24 mm) and posteriorly below the small pectoral (13 × 9 mm) and the large left pectoral (7 × 12 mm) (**Figure 1B**).

A bone CT scan confirmed the presence of a suspected area with hyper-absorbing hemline in the left part of the sternal manubrium, in correspondence with the known mediastinal neoformation described in the CT scan performed (**Figure 1C**).

**Figure 2:**
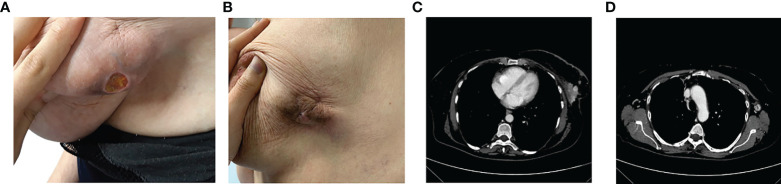
After the II cycle, the ulcerated was decreased **(A)**, up to the III cycle when there was the cutaneous resolution of the ulcerated mass **(B)**, with a partial response at the CT scan **(C)** and a complete response of pathological lymph nodes **(D)**.

Therefore, in June 2020 she underwent palliative radiotherapy (RT) of the sternum and then, after electrocardiography and signing of informed consent, she started a first line with ribociclib plus fulvestrant at the standard dosage. Already after the II cycle, there was a remarkable improvement in the cutaneous disease with a reduction of the underlying tumefaction (**Figure 2A**), up to the resolution of the ulceration after the III cycle of treatment (**Figure 2B**).

In September 2020, a CT scan showed a partial response with a significant reduction in dimension at the left QQEE (4.1 × 2.5 cm versus 10 × 6 cm of DM), skin always ulcerating (**Figure 2C**). Complete response of pathological lymph nodes was previously reported in the left internal mammary chain as well as a significant reduction of axillary adenopathies (13 versus 24 mm (**Figure 2D**). According to the objective radiological response, the treatment is still ongoing with good tolerability.

### Case Report 2: Patient With a Neodiagnosis of Locally Advanced, Inoperable HR+, HER2- Breast Cancer

A 67-year-old patient, with negative family history for oncological diseases, smoker of about 10 cigarettes/day for 20 years, affected with arterial hypertension and depression syndrome in pharmacological treatment, showed a neodiagnosis of HR+, HER2-, ABC.

**Figure 3:**
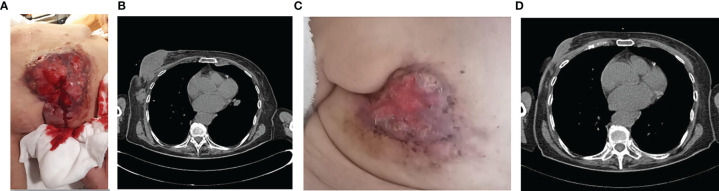
Clinico-radiological presentation of solid, cancer mass of the breast at the diagnosis **(A, B)**, and after 5 months of treatment **(C, D)**.

In March 2020, at the first oncological examination she reported a new solid mass in the right breast of about 12 cm, ulceration, and bleeding (**Figure 3A**) with tumefactions of axillary lymph nodes (DM = 5 cm) and subcutaneous satellite nodules. Right-breast biopsy diagnosed an IDC, ER = 90% PR = 90% ki67 = 20% HER2 = 1+ at the IHC evaluation.

In April, the CT scan described the presence of a large solid lesion in the right breast of 129 × 42 × 50 mm that infiltrates posteriorly the pectoral muscles, and two lymphadenopathies of 5 × 3 cm in diameter along the axillary extension with no other secondarisms (**Figure 3B**).

The patient also underwent a bone CT scan, which confirmed the absence of any bone lesions, and a tumor marker blood test (CEA = 18 ng/ml Ca15.3 = 95 U/ml). For the locally advanced, inoperable disease, in May 2020 she started a first-line treatment with ribociclib and fulvestrant. During treatment, she reported clinical response with evidence of more than 30% of reduction in the dimension of the tumor in the right breast (**Figure 3C**). Taking into account the further radiological response (**Figure 3D**), the local breast unit discussion concluded that surgery of primary tumor with flap reconstruction became amenable. Thus, the patient underwent mastectomy followed by ribociclib and fulvestrant resumption, which is still ongoing.

### Case Report 3: Patient With *De Novo* Bone Oligometastatic Disease Treated With Surgery and Radiotherapy

A 52-year-old premenopausal patient, ex-smoker, with no family history of breast or ovarian cancer, affected with arterial hypertension in pharmacological treatment, reported a diagnosis of oligometastatic breast cancer. In March 2019, she was admitted in our Emergency Department for the onset of acute pain in the left upper limb after an accidental fall at home.

**Figure 4:**
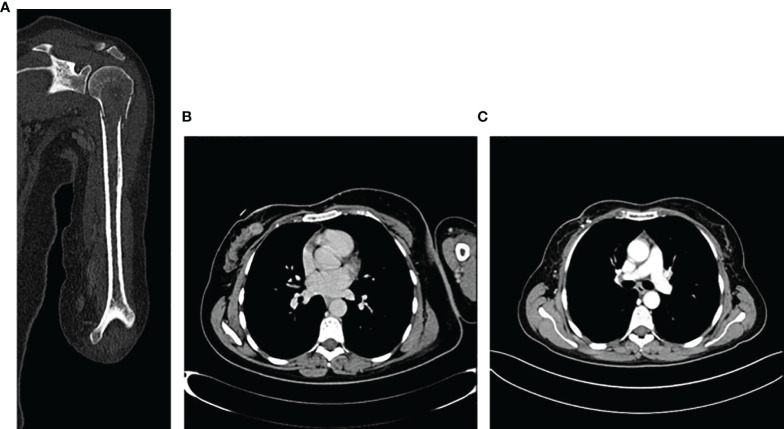
Imaging of bone lesion in the left homerus **(A)**, and the locally advanced breast cancer at the time of diagnosis in March 2019 **(B)** and after 6 months of treatment **(C)**.

The radiograph shows the presence of a pathological fracture of the left humerus with an associated suspected bone lesion. A breast ultrasound revealed a nodular formation of about 20 mm in the right upper-internal quadrant and at least three pathological lymph nodes in the ipsilateral axilla. The biopsy confirmed the diagnosis of ductal breast carcinoma G2 ER 98% PR 98% HER2 negative, Ki-67 10%. The staging procedure with a CT scan and a bone scan confirmed the presence of a LABC with a single bone metastasis in the left humerus (**Figures 4A, B** **)**. In April 2019, a bone biopsy was performed and the histological exam showed breast cancer cells with a profile ER 98% PR 80% HER2 1+ Ki67 8%. No surgical indication was given in consideration of the presence of reparative phenomena, the mild controlled pain, and the availability of active systemic treatments.

Therefore, in May 2019, she started a first-line treatment with ribociclib 600 mg for 21 days/28-day cycle plus letrozole 2.5 mg/die plus triptorelin 3.75 mg every month. After the first 3 months, the patient had a clinical benefit and no pain in the left arm and the clinical examination revealed a significant reduction of the right breast lesion. A breast ultrasound showed a significant reduction of the target breast lesion (from 20 to 10 mm) and lymph nodes. The patient continued CDK4/6i plus letrozole. At 6 months, a CT scan showed a significant reduction of primary breast cancer (**Figure 4C**) and the single bone lesion with an overall partial response. No significant toxicity was observed, and the patient continued CDK4/6i + IA. In November 2020, after 18 months of treatment, CT scan confirmed the reduction of primary cancer and a stability of the bone lesion. The case was discussed in the breast multidisciplinary unit: considering objective response and the presence of a single bone lesion, it was decided for surgery on primary tumor and radiotherapy on the left humerus metastasis. On December 15th, the patient underwent right lumpectomy plus axillary lymph node dissection (ALND). The histological examination showed 19 × 10 mm IDC with 4/6 metastatic lymph nodes G2 ER 98% PR 0 Her2 0/1+ Ki67 2% pT1c pN2a (4/6). After 20 days, the patient resumed CDK4/6i + IA and triptorelin. In February 2021, a definitive 30-Gy radiotherapy on the single bone lesion was performed with concomitant CDK 4/6i + AI. The patient, after 32 months, is currently still on first line treatment with ribociclib + letrozole + triptorelin with no evidence of locoregional recurrence or systemic disease progression. The treatment was well tolerated during the entire period.

### Case Report 4: Patient With *De Novo* Advanced Breast Cancer, With Oligometastatic Bone Disease

A 67-year-old patient, affected with arterial hypertension controlled by pharmacological treatment, cancer familiarity positive due to her father who died of gastric cancer at the age of 67 years and a grandson with a history of breast cancer, came to our attention for a neodiagnosis of ABC.

Her oncological history dates back to June 2019 when she underwent a screening mammography which revealed the presence of two contiguous thicknesses of 30 and 20 mm, respectively, with irregular margins in the external quadrants (QEE) of the right breast. Therefore, she underwent a mammary ultrasound which highlighted a hypoechoic formation of 3 cm in the QEE of the right breast with a 2-cm satellite nodule and one lymphadenopathy of 20 mm strongly suspected for localization of disease at the ipsilateral axilla.

A magnetic resonance of the breast confirmed a locally advanced breast cancer also with striae of contact with the pectoral muscle and a focal area of vascularization in a single left rib.

The breast biopsy diagnosed an IDC, moderately differentiated, ER 90%, PR 55%, Ki67 30%, HER2 1+ on IHC evaluation. The cytological exam of axillary lymph node also showed a positive result for cancer.

The objective examination confirmed the presence of a solid mass of about 4.5 cm in diameter, in the QEE right breast cancer, and the ipsilateral lymphadenopathy of at least 2 cm.

In July 2019, a CT scan demonstrated the presence of multifocal vascularized areas in the right breast cancer with several lymphadenopathies in the ipsilateral axilla and bone rearrangement with cortical interruption at the level of the right iliac wing and at the level of the 10th left rib, highly suggestive for secondary lesions.

Thus, a bone CT scan was done, and the bone involvement was confirmed.

**Figure 5:**
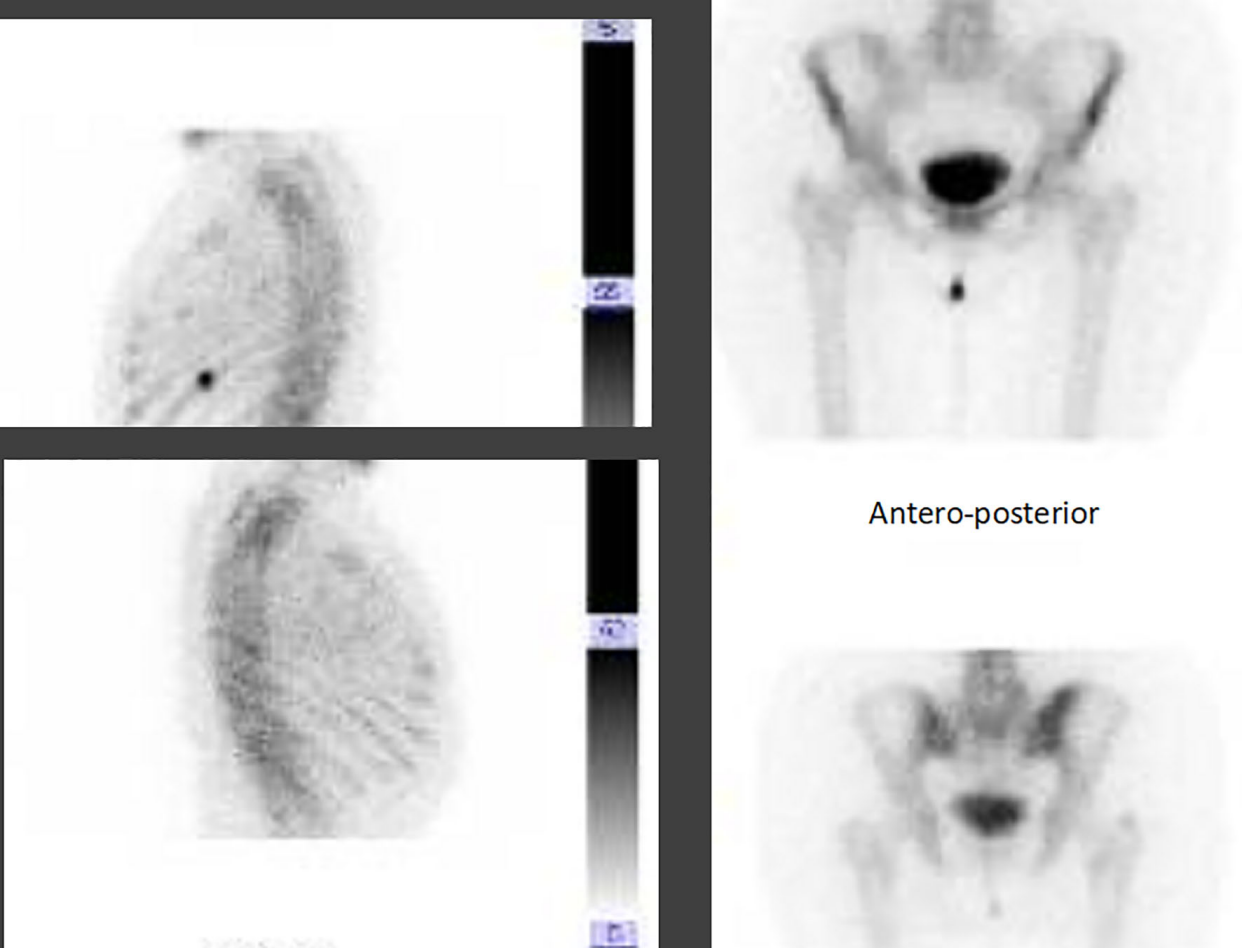
Bone scan evaluation, before and after treatment with ribociclib and letrozole.

According to the advanced disease stage, in August 2019 she started first-line treatment with ribociclib plus letrozole with the adjunct of a bisphosphonate to prevent skeletal events. However, due to bone pain being non-responsive to painful drugs, in September 2019, the patient also underwent a palliative stereotactic radiotherapy of the left iliac wing and the 10th rib (3,000 cGy on each site), with immediate pain improvement. Overall, the treatment was well tolerated, and only G1 nausea, unspecific osteomuscular pain, and G1 hypercholesterolemia were reported. In December 2019, at the first clinico-radiological evaluation partial response of both breast and axilla localizations, even with the persistency of a satellite nodule, as well as a significant reduction in bone lesions at the bone scan, was reported (**Figure 5**).

In May 2020, an 18FDG-PET CT showed a mild pathological expression of glucidic metabolism in correspondence to bone rearrangement areas with mixed characteristics to the level of the right iliac wing (SUV 2.1) and to the level of the 10th left rib (SUV 2.5). After 4 months, a further reduction in the dimension of breast cancer as well as ipsilateral lymphadenopathies, with no more evidence of satellite nodules, was reported at both mammary ultrasound and breast resonance imaging. Moreover, the 18FDG-PET CT was negative for secondary lesions. Subsequently, in December 2020 the local breast unit choice was surgery of primary tumor and maintenance of systemic endocrine treatment which is still ongoing with good tolerability.

## Discussion

Combination of CDK4/6 inhibitors plus hormone therapy represents the new standard of care in patients affected with HR+, HER2-, ABC. Indeed, CDK4/6 inhibitors with AI or fulvestrant have significantly improved the outcome of patients in terms of response rate and survival, regardless of the extension of the disease and the presence of visceral metastasis or bone-only disease. Despite the wide use of these combinations in all the subgroups of HR+, HER2- ABC, literature data about patients with locally advanced, inoperable disease are still poor and it is even less known what oncologists have to do in case of complete radiological response after locoregional treatment in patients with an oligometastatic condition.

We present case reports of four patients treated with ribociclib plus ET as first-line therapy for i) a locally advanced, inoperable HR+, HER2- relapse; ii) a neodiagnosis of locally advanced, inoperable disease; iii) a *de novo* bone oligometastatic disease treated with surgery and radiotherapy; and iv) a neodiagnosis of ABC with low-volume, bone-only disease.

In the first case, we report a patient with an endocrine-sensitive ABC disease, who relapsed after 10 years form the first diagnosis and reported a locally ABC with an enlarged, ulcerated, inoperable, breast cancer mass close to the sternum, which was infiltrated. Thus, patients underwent radiotherapy to the local control and started ribociclib plus fulvestrant as systemic therapy. The treatment was very well tolerated, and just after the third cycle the patient reported a complete remission of distant metastatic disease with a partial response of breast tumor, potentially amenable for primary surgery. The second case was a *de novo* ABC, with no distant metastases, treated with ribociclib and fulvestrant, resulting in a significant response in the breast primary tumor. Similar to a neodjuvant setting, after the maximum effect in the local disease was obtained, the patient underwent surgery of primary tumor and now she has no evidence of disease and continued systemic ET to lower the risk of relapse.

As reported in literature, HR+, HER2-, with low ki67, breast cancer have not been completely satisfactory response rates to standard neoadjuvant chemotherapy (13). Therefore, as suggested by our cases, the hormone-based strategy as primary treatment in locally advanced, inoperable diseases could be considered as a significant option not only in patients considered unfit for chemotherapy but also in all those patients who were affected by a low-proliferative, HR+, HER2-, breast cancer. Moreover, due to the lack of data in MONALEESA trials, with the exception of a single-case report (14), which described complete response of *de novo* breast cancer treated with CDK 4/6 inhibitors in combination with endocrine therapy, our case report is of great interest for the scientific community.

Particularly, combinations of ET with cdk4/6-targeted treatment could ensure great results in these patients. Indeed, several studies which explored the role of ET plus CDK4/6 inhibitors as neoadjuvant treatment are still ongoing in patients with a neodiagnosis of HR+, HER2-, LABC with promising results (15–19) and delayed quality-of-life deterioration (20). The recent phase II trial CORALLEEN in patients with high-risk luminal B breast cancer demonstrated response rates similar to multiagent chemotherapy with ribociclib/letrozole but with less toxicity (21). However, the demonstrated clinical efficacy of ribociclib and the other CDK4/6 inhibitors in metastatic settings cannot yet be translated in higher pathological complete response in neoadjuvant settings, where the role of targeted agents is still debated (22).

Despite data being still debated, it has been demonstrated that patients who reported a decrease in ki67 evaluation according to an antiproliferative effect showed better results. Thus, it has been suggested that Ki67 decreased expression not only as prognostic factor (23) but maybe also as predictive of response to ET plus CDK4/6i target treatment.

The third and fourth cases reported patients with HR+, HER2- ABC with low-volume, bone oligometastatic disease. In both cases, the patients obtained a significant response to first-line treatment with ribociclib and letrozole, with no more evidence of disease. Specifically, in the last case due to complete remission of bone disease, and the locoregional objective response, according to literature data on the role of surgery for primary tumor in metastatic patients (24, 25), the local breast unit decided for breast mastectomy, maintaining systemic treatment even if in the absence of evidence of distant disease. Indeed, we do not know what oncologists have to do in these cases of initially oligometastatic disease which reported a complete remission after treatment (stop treatment? Stop only CDK4/6 inhibitors and maintain AI? And how long)?. According to the promising data of abemaciclib in terms of improving interval disease-free survival (iDFS) in adjuvant settings, indirectly translating these results in cases of patients who obtained complete remission, we could also consider maintaining CDK4/6 inhibitors for 2 years (26, 27). Cancer progression is a multistep process, and at the oligometastatic stage the cancer’s full metastatic potential has not yet reached, limiting it to certain sites in the body that are more receptive, implicating the “seed and soli” theory (28). Actually, the microRNA profiling expression of metastatic cells revealed that the oligometastatic phenotype seems to be a genetically distinct entity rather than just a “transition point” from primary tumor to widespread metastasis (29). Several studies, evaluating radical treatment of oligometastatic breast cancer, have shown conflicting results in terms of management of the primary tumor, although outcomes are more promising for the management of metastatic disease, especially when it was reported in liver or lung parenchyma. Furthermore, in the most recent clinical trials evaluating new systemic therapies, radical management of the primary tumor or metastatic lesions was not allowed, making it difficult to gain a precise understanding of the percentage of patients who may reach a no-evidence-of-disease status with the current standards of care for ABC (30). Of note, some studies suggest that in case of complete remission of the disease as in patients with early stage, surgery of primary tumor should be considered. However, to date the removal of the primary tumor in patients with *de novo* stage IV breast cancer has not been associated with survival improvement, with the possible exception of the subset of patients with bone-only disease. Furthermore, surgery of primary tumor should be considered in selected patients, with controlled systemic disease, or low-volume, oligometastatic disease that is highly sensitive to systemic therapy, particularly to improve quality of life, always taking into account the patient’s preference (1). However, data about the outcomes of oligometastatic endocrine-sensitive patients treated with CDK4/6 inhibitors followed by locoregional treatments with no more evidence of disease, are still lacking. According to recent literature data (31, 32), our results demonstrated that the radical approach to oligometastatic disease should always be considered in the course of the cancer history, mainly during the first-line therapy. Indeed in patients with a controlled primary tumor and one to five oligometastatic lesions, a stereotactic ablative radiotherapy (SABR) could be a great strategy combined with the standard palliative care to improve survival, oncological outcomes, toxicity, and quality of life (33). According to the limit of a case-series article, we conclude that those patients who achieve a complete radiological response during first-line treatment with the CDK4/6 inhibitor should always be evaluated in a breast unit for radical treatment of primary tumor and/or locoregional treatment of metastatic lesions to significantly improve their outcome.

## Conclusion

We reported data of patients treated with ribociclib and ET as first-line therapy for a locally advanced or oligometastatic ABC and who underwent a curative locoregional treatment wherever possible, aimed at achieving a complete remission status. Overall, our data of ribociclib as primary treatment are really positive and all our four patients are still under treatment with a favorable safety profile. Despite these promising results, the question about what we have to do in those patients with no evidence of disease during CDK4/6 inhibitor or after locoregional curative treatments is still unknown. Thus, additional, large-sample retrospective and/or prospective trials evaluating the role of CDK4/6 inhibitor in these settings are awaited.

## Data Availability Statement

The raw data supporting the conclusions of this article will be made available by the authors, without undue reservation.

## Ethics Statement

Ethical review and approval were not required for the study on human participants in accordance with the local legislation and institutional requirements. The patients/participants provided their written informed consent to participate in this study. Written informed consent was obtained from the individual(s) for the publication of any potentially identifiable images or data included in this article.

## Author Contributions

AB, AF, and PM contributed to the conception and design of the study. MR wrote the manuscript. All authors contributed to the manuscript revision and read and approved the submitted version.

## Conflict of Interest

The authors declare that the research was conducted in the absence of any commercial or financial relationships that could be construed as a potential conflict of interest.

## Publisher’s Note

All claims expressed in this article are solely those of the authors and do not necessarily represent those of their affiliated organizations, or those of the publisher, the editors and the reviewers. Any product that may be evaluated in this article, or claim that may be made by its manufacturer, is not guaranteed or endorsed by the publisher.
